# An Efficient Method for Immortalizing Mouse Embryonic Fibroblasts by CRISPR-mediated Deletion of the *Tp53* Gene

**DOI:** 10.21769/BioProtoc.5159

**Published:** 2025-01-20

**Authors:** Srisathya Srinivasan, Hsin-Yi Henry Ho

**Affiliations:** Department of Cell Biology and Human Anatomy, University of California, Davis, School of Medicine, Davis, CA, USA

**Keywords:** Mouse embryonic fibroblasts (MEFs), Immortalized mouse embryonic fibroblasts (iMEFs), MEF isolation, MEF immortalization, *Tp53* knockout, CRISPR, Electroporation

## Abstract

Mouse embryonic fibroblasts (MEFs) derived from genetically modified mice are a valuable resource for studying gene function and regulation. The MEF system can also be combined with rescue studies to characterize the function of mutant genes/proteins, such as disease-causing variants. However, primary MEFs undergo senescence soon after isolation and passaging, making long-term genetic manipulations difficult. Previously described methods for MEF immortalization are often inconsistent or alter the physiological properties of the cells. Here, we describe an optimized method that overcomes these limitations. By using electroporation to deliver CRISPR constructs that target the *Tp53* gene, the method reliably generates immortalized MEFs (iMEFs) within three weeks. Importantly, iMEFs closely resemble the parent cell populations, and individual iMEFs can be cloned and expanded for subsequent genetic manipulation and characterization. We envision that this protocol can be adopted broadly to immortalize other mouse primary cell types.

Key features

• CRISPR-based knockout of the *Tp53* gene enables efficient immortalization of mouse embryonic fibroblasts (MEFs) in under three weeks.

• Immortalization requires a Neon electroporator or a comparable system to transfect cells with the *Tp53* CRISPR constructs.

## Background

The ability to culture cells long-term in vitro has been a cornerstone of modern cell biological research. Except for stem and cancer cells, most primary cells undergo senescence in culture, making immortalization an essential step during the establishment of permanent cell lines from native tissues. Earlier methods of cell immortalization involved overexpression of oncogenes to transform cells [1–4]. Though efficient, these methods frequently result in the acquisition of cancer-like phenotypes, including the loss of contact inhibition and anchorage-dependent growth, as well as alterations in growth factor requirement, metabolism, and other signaling activities [5–7].

Overexpression of telomerase (TERT, or telomerase reverse transcriptase) is another common method to transform mammalian cells [8]. This method is highly effective in immortalizing human cells and has a lower tendency to induce cancer-like phenotypes [9]. However, few examples exist in the literature demonstrating that TERT overexpression alone can immortalize mouse cells [10], suggesting that this method is generally less effective toward mouse cells. In our own experience, we have not been able to successfully immortalize mouse embryonic fibroblasts via overexpression of telomerase.

3T3 cells are widely used mouse embryonic fibroblast (MEF) lines spontaneously immortalized through serial passaging. The “3T3” method, originally described by Howard Green and colleagues, involves seeding primary MEFs at 3 × 10^5^ cells per 50-mm dish and transferring every 3 days at the same density until rapidly dividing, immortalized cells emerge [11]. While this immortalization process is considered gentle and does not cause neoplastic transformation [11], it is inefficient and time-consuming. Moreover, because the method relies on mutation(s) that spontaneously arise during the prolonged cell passaging phase, it is difficult to compare cell lines derived from different immortalization experiments.

Notably, Harvey and Levine [12] reported that MEF lines immortalized via the 3T3 method frequently carry loss-of-function mutations in the tumor suppressor gene *Tp53*, suggesting that this is a key immortalizing event. Consistent with this observation, we found that CRISPR-mediated ablation of the *Tp53* gene robustly immortalizes primary MEFs. We have used the protocol described here to reliably generate immortalized MEF lines (iMEFs) in under three weeks and from as few as 50,000 primary cells. iMEF lines have also been successfully established from wildtype (WT) and genetically modified embryos of different genetic backgrounds (C57BL/6 and mixed C57BL/6; 129J). Lastly, iMEFs generated through *Tp53* deletion can be subcloned and genetically manipulated further in downstream applications, such as gene rescue experiments.

## Materials and reagents

**Biological materials**

1. Pregnant mice carrying embryonic day 12.5 (E12.5) embryos

**Reagents**

1. Phosphate buffered saline (PBS) without Ca^2+^ and Mg^2+^ (Cytiva, catalog number: SH30256.01)

2. Hanks’ balanced salt solution (HBSS) without Ca^2+^ and Mg^2+^ (Gibco, catalog number: 14170112)

3. Trypsin from bovine pancreas (Worthington Biochemical, catalog number: LS003702) for MEF preparation

4. RQ1 RNase-free DNase (1 u/μL) (Promega, catalog number: M6101)

5. Non-essential amino acids solution in minimal essential medium (MEM NEAA) (100×, Gibco, catalog number: 11140050)

6. Sodium pyruvate (100 mM) (Gibco, catalog number: 11360070)

7. Dulbecco’s modified Eagle’s medium (DMEM), high glucose (4.5 g/L), 1× (Gibco, catalog number: 11960-044)

8. Fetal bovine serum (FBS), United States qualified grade (Gibco, catalog number: 26-140-079; this product has been discontinued, but other similar grade FBS can be used)

9. Penicillin-streptomycin (100×) (Gibco, catalog number: 15-140-122)

10. L-glutamine (200 mM) (Gibco, catalog number: 25030081)

11. Dimethyl sulfoxide (DMSO), sterile, tissue culture grade (Millipore Sigma, catalog number: D2650-100ML)

12. 0.25% trypsin-EDTA with phenol red (Gibco, catalog number: 25200072), for cell harvest and passaging after the initial MEF preparation step

13. Px461-Cas9n-Trp53-sgRNA-alpha plasmid (Addgene, plasmid number: 88846; generated and deposited by the Massagué lab) [13]

14. Px461-Cas9n-Trp53-sgRNA-beta plasmid (Addgene, plasmid number: 88847; generated and deposited by the Massague lab) [13]

15. pCAG-GFP plasmid (Addgene, plasmid number: 11150; generated and deposited by the Cepko lab) [14]

**Solutions**

1. 0.1% trypsin for MEF preparation (see Recipes)

2. Complete cell culture media (culture media) (see Recipes)

3. Cell freezing media (see Recipes)

**Recipes**

**1. 0.1% trypsin for MEF preparation**

**Table:** 

Reagent	Final concentration	Amount
Trypsin from bovine pancreas	0.1%	40 mg
HBSS	1×	40 mL

Filter sterilize with a 0.22 μm syringe filter. Aliquot, snap freeze in liquid nitrogen, and store at -80 °C.

**2. Complete cell culture media (culture media)**

**Table:** 

Reagent	Final concentration	Amount
DMEM	1×	500 mL
FBS	10%	50 mL
Penicillin-streptomycin (100×)	1×	5.5 mL
L-glutamine (200mM)	2mM	5.5 mL

Prewarm at 37 °C before use. For culturing freshly isolated MEFs (steps A2k–o), supplement culture media with sodium pyruvate (1mM final) and MEM NEAA (1× final). For culturing cells immediately after electroporation (steps B3–19), use culture media without penicillin-streptomycin.

**3. Cell freezing media**

**Table:** 

Reagent	Final concentration	Amount
Culture media	90%	45 mL
DMSO	10%	5 mL

Filter sterilize with a 0.22 μm syringe filter.

**Laboratory supplies**

1. Surgical scissors (Fine Science Tools, model: Surgical scissors-sharp, catalog number: 14002-12)

2. Adson forceps (Fine Science Tools, model: Adson forceps, catalog number: 11006-12)

3. Fine tip forceps (Fine Science Tools, model: Dumont #5 Inox, catalog number: 11251-20)

4. 60 mm tissue culture (TC)-treated cell culture dishes (Corning, Falcon, catalog number: 353002)

5. 100 mm TC-treated cell culture dishes (Corning, Falcon, catalog number: 353003)

6. 6-well clear TC-treated multiple well plates (Corning, Costar, catalog number: 3516)

7. 24-well clear TC-treated multiple well plates (Corning, Costar, catalog number: 3524)

8. 50 mL conical centrifuge tubes (Thermo Fisher Scientific, Nunc, catalog number: 339653)

9. Pipettes (Eppendorf, Research Plus, P-1000, P-200, P-20, catalog number: 3123000918)

10. Aerosol barrier pipette tips [Fisher Scientific, SureOne, catalog number: 02-707-442 (0.1–10 μL), 02-707-432 (2–20 μL), 02-707-430 (20–200 μL), 02-707-404 (100–1,000 μL)]

11. Metal tube rack for 1.5 mL tubes (Stratagene, catalog number: 41018; the item has been discontinued, but any similar rack will work)

12. 5 mL serological pipettes (Thermo Fisher Scientific, Nunc, catalog number: 170355N)

13. Pipette controller (Drummond, Pipet-Aid, catalog number: 4-000-100)

14. 1.5 mL microcentrifuge tubes (Denville Scientific, PosiClick, catalog number: C2170)

15. Neon transfection system 10 μL kit (Thermo Fisher Scientific, Neon, catalog number: MPK1096); includes Neon tips, Neon tubes, buffer R, and buffer E

16. TransIT-LT1 transfection reagent (Mirus Bio, catalog number: MIR 2304)

17. Cell strainers (Corning, Falcon, catalog number: 352340)

18. 30 mL syringes (BD, catalog number: 302832)

19. 0.22 μm syringe filters (Thermo Fisher Scientific, Fisherbrand, catalog number: 09-719C)

20. Cryogenic vials (Corning, catalog number: 430488)

## Equipment

1. Dissecting microscope (Leica, model: S7E, catalog number: S7E-PS)

2. Biological safety cabinet (Thermo Fisher Scientific, model: 1300 series class II, catalog number: 1323TS)

3. CO_2_ incubator (Thermo Fisher Scientific, model: Heracell 150i, catalog number: 51026281), set at 37 °C with 5% CO_2 _and 90%–95% humidity

4. Benchtop centrifuge (Thermo Fisher Scientific, model: Sorvall ST 8, catalog number: 75007200)

5. 8 × 50 swinging bucket rotor (Thermo Fisher Scientific, model: TX-100S, catalog number: 75005704)

6. Microcentrifuge (Thermo Fisher Scientific, model: Sorvall Legend Micro 21, catalog number: 75002436)

7. 24 × 1.5/2.0 mL rotor with ClickSeal lid (Thermo Fisher Scientific, catalog number: 75003424)

8. Ultra-low (-86 °C) freezer (Thermo Fisher Scientific, model: Revco UxF, catalog number: UXF60086A; this model has been discontinued)

9. Liquid nitrogen storage unit (Thermo Fisher Scientific, model: Cryoplus 3, catalog number: 7404)

10. Cell counter (Corning, catalog number: 6749)

11. Electroporation device and Neon pipette (Thermo Fisher Scientific, model: Neon, catalog number: MPK5000. This instrument has been discontinued; other similar electroporation devices can be used, but optimization will be required.)

12. Inverted fluorescence microscope (Thermo Fisher Scientific, model: Evos FL, catalog number: AMF4300)

13. Mr. Frosty freezing container (Thermo Fisher Scientific, catalog number: 5100-0001)

## Procedure

**A. Preparation of primary mouse embryonic fibroblasts (MEFs)**

*Performing the embryo dissection in a laminar flow hood is ideal. However, if a laminar flow hood is not available, the procedure can be performed on a standard lab bench. All solution stocks (e.g., HBSS) should be kept sterile and transferred to the dishes used for dissection in a biosafety cabinet. General procedures on mouse husbandry can be found in [15].*

1. Dissection of E12.5 embryos from timed mating

a. Set up timed mating and start checking the females for vaginal plugs the following morning.

**Figure 1.**
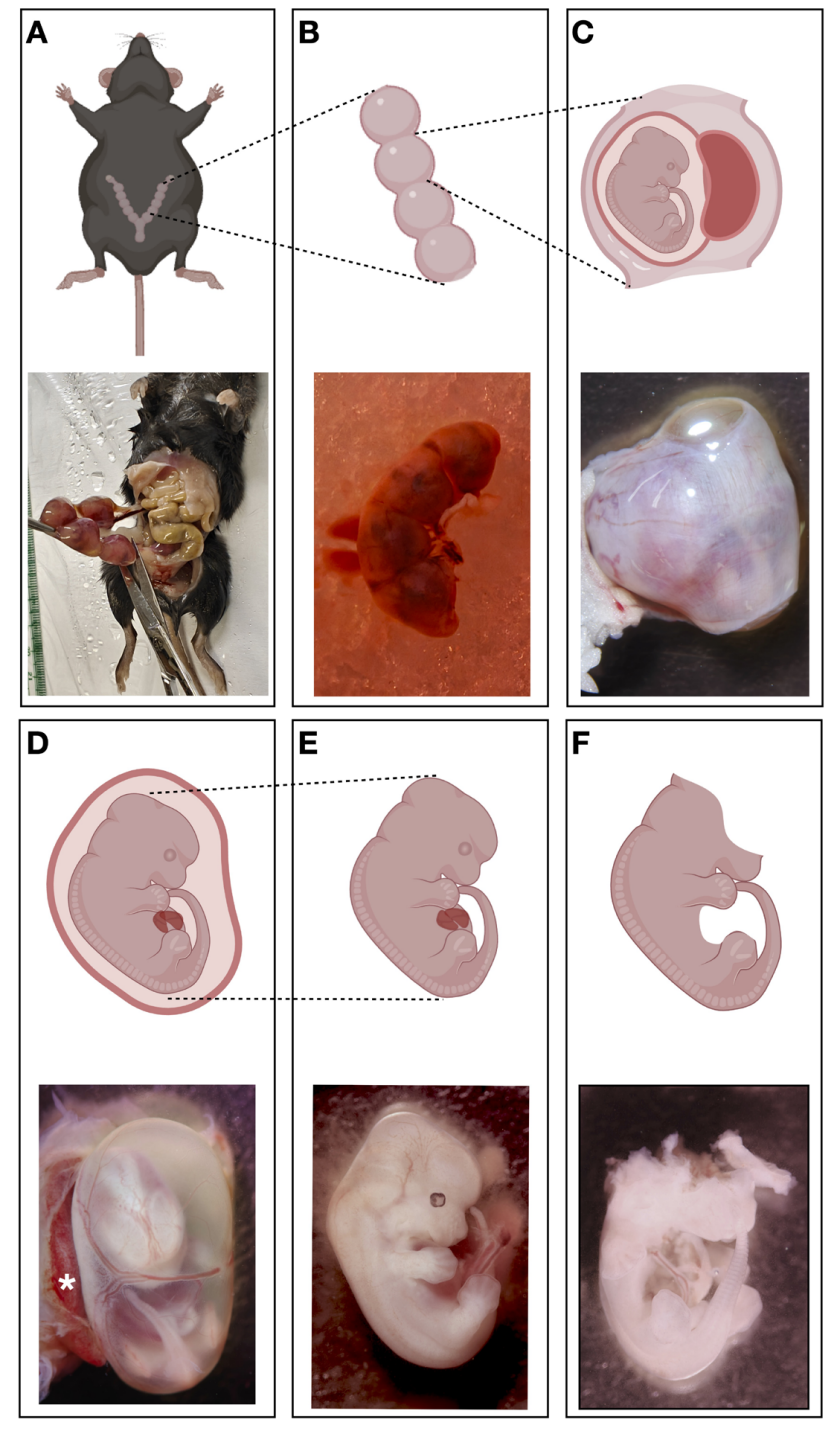
Key steps of embryo dissection. A. Top panel: the approximate position of the uterus in the dam. Bottom panel: dissected dam showing the uterus with embryos. B. A dissected uterine horn with embryos in it. C. An embryo still wrapped in the transected uterine wall. D. An embryo still encased in the embryonic sacs. * denotes the placenta. E. An embryo after the removal of the embryonic sacs and placenta. F. An embryo after the removal of the brain, eyes, and internal organs.

**Critical**: Plugged females should be separated from the male and housed until 12.5 days post-coitum.

b. Euthanize the dam at 12.5 days post-coitum via CO_2 _inhalation followed by cervical dislocation to ensure death.

c. Spray the dam with 70% ethanol to sterilize the abdominal surface.

d. Dissect out the uterine horns and rinse in PBS in 100 mm dishes (Figure 1A–1B).

**Critical**: When dissecting the uterine horns, ensure that the intestines are not nicked and the uterine horns do not touch the fur to prevent contamination.

e. Separate the embryos from the uterus and place each embryo in a 60 mm dish containing HBSS (Figure 1C–1D).

f. In the same 60 mm dish and under a dissecting microscope, remove the placenta and embryonic sac (Figure 1D–1E).

*Note 1: The embryonic sacs can be collected and used to genotype the embryos. One-half to one-quarter of each sac is sufficient.*

*Note 2: The sac should be gripped firmly with fine-tip forceps and rinsed briefly under a slow stream of running deionized water before transferring to a collection tube. This helps to prevent cross-contamination from maternal tissues.*

g. Remove the brain, eyes, and internal organs of the embryo as much as possible using fine-tip forceps and discard them (Figure 1E–1F).

h. Transfer the dissected embryo to a 6-well plate filled with HBSS and keep the plate on ice until all embryos have been dissected. Place one dissected embryo in each well of the 6-well plate.

**Critical**: The plate(s) should be kept on ice to ensure the viability of embryonic tissues. Proceed to step A2 as soon as possible.

*Note: The carcass of the dam and other maternal and embryonic tissues should be disposed of through approved biohazard protocols.*

2. Preparation of MEFs

*This and all subsequent steps should be performed in a biosafety cabinet under sterile conditions.*

Before starting:

- Prewarm the metal rack at 37 °C in the TC incubator.
- Thaw 0.1% trypsin (from bovine pancreas) at 37 °C and keep it on ice once fully thawed.
- Label 1.5 mL microcentrifuge tubes for embryo homogenization. Label as many tubes as there are embryos.
  a. Add 25 μL of RQ1 DNase into the first labeled 1.5 mL tube. Add 0.5 mL of 0.1% trypsin to the same tube and mix by pipetting 2–3 times with a P1000 pipette.
  b. Transfer the first embryo from the 6-well with HBSS into the tube (containing trypsin and DNase I) using a P1000 pipette while applying gentle suction.
  c. Homogenize the embryo by pipetting up and down 12–15 times using a P1000 pipette until the embryo is broken into small chunks (Figure 2A).
  d. Store the tube with the homogenized embryo on ice and proceed to homogenize the next embryo (repeating steps A2a–A2c), until all embryos are homogenized.

**Critical**:

- Prepare each tube of RQ1 DNase and trypsin after processing the previous embryo. This ensures optimal activity of the DNase.
- It is very important to not over-homogenize the embryo as this can cause excessive cell lysis. When the homogenate starts to feel increasingly viscous, it is over-homogenized.
  e. Transfer the tubes with the homogenized embryos to the prewarmed metal rack and incubate at 37 °C for exactly 8 min.
*Note: The homogenized tissue chunks tend to settle down in the tube. Invert the tube every ~3 min to mix the contents of the tube.*
  f. While the incubation is ongoing, add 5 mL of cold culture media to a 50 mL conical tube in preparation for the next step. Prepare as many tubes as there are embryos.
*Note: The media should be cold to facilitate rapid neutralization of the trypsin.*
  g. After the 8-min incubation at 37 °C, transfer 1 mL of the cold culture media from the 50 mL tube (step A2f) to the 1.5 mL microcentrifuge tube containing the homogenate and trypsin (Figure 2B) to neutralize the trypsin.
  h. Pipette gently 2–3 times with a P1000 pipette and transfer the contents (Figure 2C) into the 50 mL tube with the remaining 4 mL cold culture media (from step A2f).
  i. Use a 5 mL serological pipette to pipette the mixture up and down 8–10 times very gently.
  j. Centrifuge the neutralized homogenates at 500× *g* for 10 min at room temperature in a centrifuge equipped with a swinging bucket rotor.
  k. While centrifugation is ongoing, add 10 mL of prewarmed culture media supplemented with 100 μL of sodium pyruvate (1 mM final concentration) and 100 μL of MEM NEAA (1× final concentration) to a 100 mm TC-treated cell culture dish. Prepare as many dishes as there are embryos. Keep the dishes in the TC incubator.
  l. Once the centrifugation is done, carefully remove the supernatant by aspiration.
**Critical**: Be careful to avoid disturbing the pellet (Figure 2D) or getting the aspirator tip too close to the pellet, as the pellet can easily get aspirated away.
  m. Gently resuspend each pellet in 1 mL of culture media supplemented with sodium pyruvate and MEM NEAA (from the 100 mm dish prepared in step A2k) using a P1000 pipette and transfer the cell suspension back to the same prewarmed 100 mm dish.
  n. Shake the plate gently to ensure uniform distribution of the cells.
  o. Incubate the cells in the TC incubator (37 °C, 5% CO_2_, 90%–95% humidity) without disturbance until the next day.
  p. The next morning, observe the cells to assess cell health (Figure 2E–2F).
*Note 1: The MEFs should be approximately 80% confluent one day after isolation and reach full confluency within 2–3 days. Some cell death is expected, but most cells should survive and adhere to the culture dish by the morning after isolation (Figure 2E).*
*Note 2: It is normal for the MEF cultures to contain clumps of tissue that are not fully dissociated (Figure 2F). Leave the clumps as they are.*
  q. Once the cells are confluent, wash once with sterile PBS and trypsinize the cells in each 100 mm dish with 3.5 mL of prewarmed 0.25% trypsin-EDTA.
  r. Once the cells lift off the plate (~1–2 min), neutralize the trypsin with 7 mL of culture media.
  s. Filter the cell suspension (still in the trypsin/culture media mixture) through a 40 μm cell strainer and collect the flowthrough in a 50 mL tube.
*Note: This step removes any remaining large cell/tissue clumps.*
  t. Centrifuge the cell suspension at 500× *g* for 5 min at room temperature in a centrifuge equipped with a swinging bucket rotor.
  u. Carefully aspirate the supernatant. At this point, cells can be split and cultured further (step A2v below) or frozen (step A2w below).
  v. To culture the MEFs further, resuspend the cell pellet in 1 mL of culture media and pipette 12–15 times with a P1000 pipette. Proceed with splitting into the desired number of dishes (ideally no more than a 1:5 split).
  w. To freeze the MEFs, resuspend the cell pellet from each 100 mm dish in 1 mL of cell freezing media and transfer to a cryogenic vial. Smaller aliquots can be prepared if desired. Put the vials in a room-temperature Mr. Frosty freezing container and then store them in an ultra-low freezer at -80 °C overnight. For long-term storage, transfer the cryogenic vials to a liquid nitrogen freezer.
**Pause point**: Primary MEFs can be stored in liquid nitrogen for many years.
**Caution**: Precautions (eye protection, cryo-safe gloves, and lab coat) must be taken while handling liquid nitrogen.
  **Figure 2.**
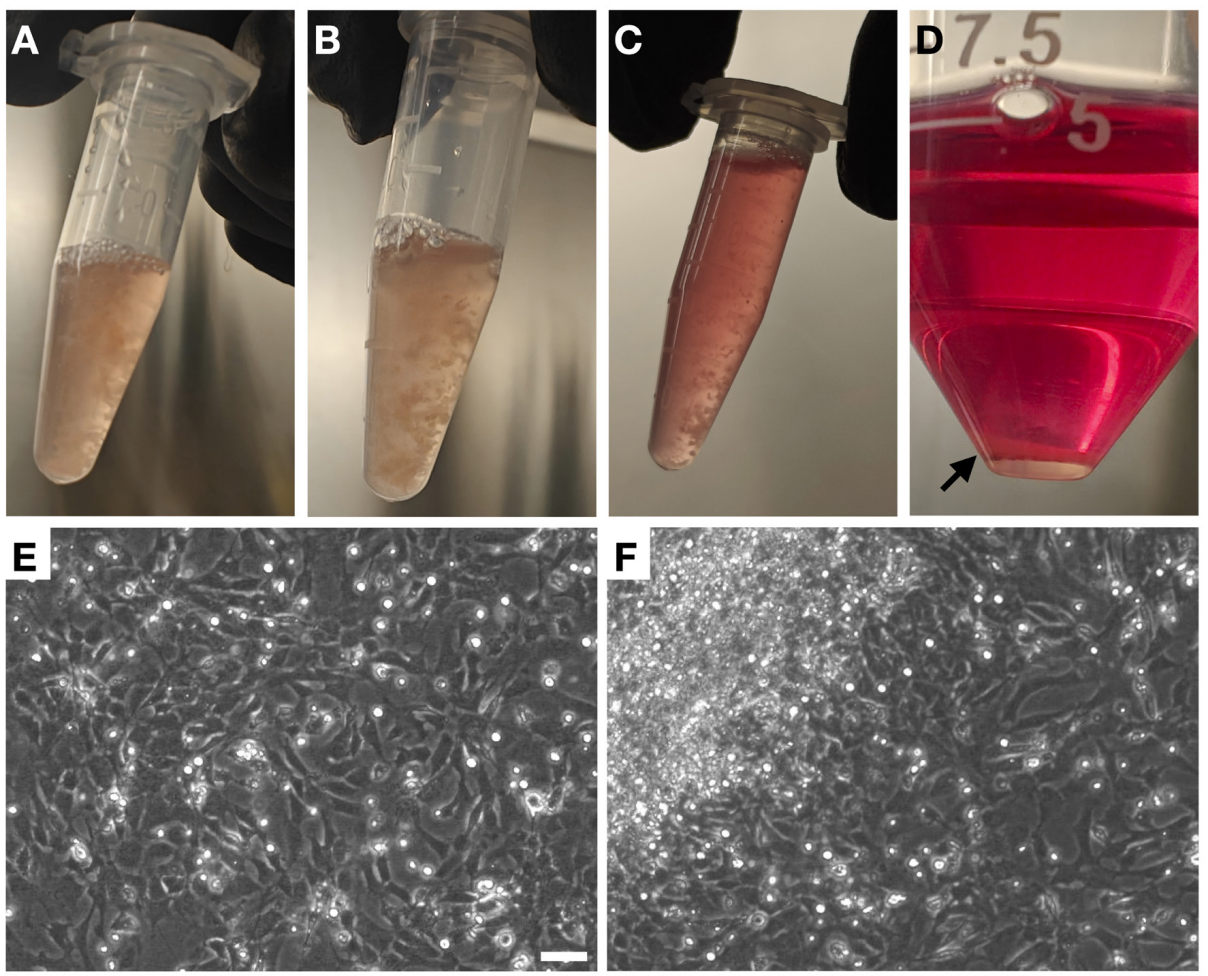
Key steps of mouse embryonic fibroblast (MEF) preparation. A. Embryo homogenate before the 8-min, 37 °C incubation with trypsin. B. Embryo homogenate after the 8-min, 37 °C incubation with trypsin. C. Embryo homogenate after neutralization of trypsin with 1 mL of culture media. D. Cell pellet (indicated by the black arrow) after centrifugation of the homogenate. E–F. Representative phase contrast images of MEFs 16 h after isolation. A large clump of cells is visible in the upper left corner of (F) Scale bar: 50 μm. Magnification: 10×.

**B. Immortalization of MEFs**

Before starting:

- Approximately 2 days before electroporation, seed a MEF stock such that cells reach 70%–90% confluency and are growing robustly on the day of electroporation. Cells can be split from an ongoing culture or thawed from a frozen stock. Cells should be under 3 passages. We routinely use passage 0 (P0) or P1 cultures. A minimum of 200,000 cells are ideal for the experiment. We typically prepare 1–2 million cells (two wells of a 6-well plate)
- Prepare the plasmids required for the immortalization procedure and adjust the concentration to 1 μg/μL.
- Before starting the electroporation experiment, prepare culture media without penicillin-streptomycin and add 500 μL per well to a 24-well plate; prewarm at 37 °C. Also, prewarm 0.25% trypsin-EDTA at 37 °C.
- Review the Neon electroporator guide (https://assets.thermofisher.com/TFS-Assets/LSG/manuals/neon_device_man.pdf; https://www.thermofisher.com/content/dam/LifeTech/migration/en/filelibrary/cell-culture/neon-protocols.par.71910.file.dat/mouse%20embryonic%20fibroblasts%20(mef)-embryo.pdf).

1. Wash cells once with PBS (1 mL for each 6-well) and trypsinize with 0.25% trypsin-EDTA (0.3 mL for each 6-well).

2. Incubate at 37 °C until the cells lift off the plate (~1–2 min).

3. Neutralize the trypsin with culture media without penicillin-streptomycin (0.7 mL for each 6-well) and transfer the cell suspension to a 1.5 mL microcentrifuge tube or another appropriately sized tube.

4. Pipette the cells 8–10 times using a P1000 pipette to achieve a single-cell suspension.

5. Count the cells and determine the cell density (cells are still in the trypsin/culture media mixture at this point).

6. Transfer 100,000 cells into each of two 1.5 mL microcentrifuge tubes (each transfection requires 50,000 cells; the two sets of cells are prepared for *Tp53* CRISPR and GFP transfections. Double the amount of cells is prepared in each set to account for pipetting errors).

7. Centrifuge the cells at 500× *g* for 5 min in a microcentrifuge. Carefully aspirate the trypsin/culture media mixture.

8. Resuspend the cell pellet in 0.5 mL of PBS. Centrifuge to pellet the cells (as in step B7).

**Critical**: This step removes the residual trypsin/culture media mixture and is critical for successful electroporation.

9. Carefully aspirate the PBS and resuspend the cell pellet in each tube in 20 μL of TransIT-LT1 transfection reagent. This is equivalent to a cell density of 5 × 10^6^ cells/mL in the transfection reagent.

*Note 1: Remove as much PBS as possible without disrupting the cell pellet, which is very small, before adding the transfection reagent.*

*Note 2: The TransIT-LT1 transfection reagent causes less cell death than buffer R included in the Neon transfection kit. However, we have had success with buffer R as well.*

10. For the *Tp53* CRIPSR and GFP control transfections, prepare a reaction mixture according to Table 1.

*Note: Each electroporation reaction requires 10 μL of the reaction mixture. To account for pipetting errors, a reaction mixture with a larger volume (20 μL) is prepared. If desired, duplicates or triplicates of the electroporation procedure can be performed to safeguard against occasional suboptimal electroporation (see note under step B16).*

**Table 1.** Recipe for the *Tp53* CRISPR and GFP reaction mixtures used for electroporation

	*Tp53* CRISPR reaction mixture	GFP reaction mixture
MEF suspension in the transfection reagent (density: 5 × 10^6^ cells/mL)	20 μL	20 μL
pCAG-GFP (1 μg/µL)	--	0.5 μL
Px461-Cas9n-Trp53-sgRNA-alpha (1 μg/μL)	0.25 μL	--
Px461-Cas9n-Trp53-sgRNA-beta (1 μg/μL)	0.25 μL	--

11. Mix the cells and DNA gently by pipetting using a P20 pipette.

12. Fill the Neon tube with 3 mL of buffer E and insert into the pipette station of the Neon transfection system.

*Note: The pipette station should be placed in the biosafety cabinet to ensure sterility. The pulse generator can be kept outside the biosafety cabinet.*

13. Insert a Neon tip into the Neon pipette by pressing the push-button on the Neon pipette to the second stop. Firmly insert the Neon pipette into the Neon tip. Gently release the push button while still applying some downward pressure to ensure a tight fit.

14. Pipette 10 μL cell/DNA suspension from the *Tp53* CRISPR reaction mixture.

Critical 1: Ensure that there are no air bubbles trapped in the tip.

Critical 2: Ensure that the cells in the master mix are in a uniform suspension. If cells have settled, mix by pipetting up and down using the Neon pipette.

15. Insert the Neon pipette into the Neon tube with buffer E.

16. Electroporate using the program below (Table 2):

**Table 2.** Electroporation parameters for MEF immortalization

Pulse voltage (V)	Pulse width (ms)	Number of pulses
1350	30	1

*Note 1: A very small spark may occur during the pulse. If a large visible spark is seen, it could indicate an issue in the conductance due to a trapped air bubble.*

*Note 2: The electroporation parameters may need to be optimized for specific experiments, especially if embryos of a different age or genetic background are used. More information on alternate programs is available in the Neon electroporator guide (* *https://assets.thermofisher.com/TFS-Assets/LSG/manuals/neon_device_man.pdf* ).

17. Immediately transfer the cells from the Neon pipette tip into the prepared 24-well plate (prewarmed culture media without penicillin-streptomycin). Gently shake the 24-well plate to evenly distribute the cells.

18. Repeat steps B14 to B17 for the GFP control construct using the GFP reaction mixture.

*Note: We routinely use each tip for up to six electroporations. However, a fresh tip can be used for each electroporation if desired.*

19. Incubate the cells in a TC incubator (37 °C; 5% CO_2_; 90%–95% humidity).

*Note: Cells can be observed for viability ~2 h after electroporation.*

**Critical**: Plate disturbance should otherwise be kept to a minimum.

**Figure 3.**
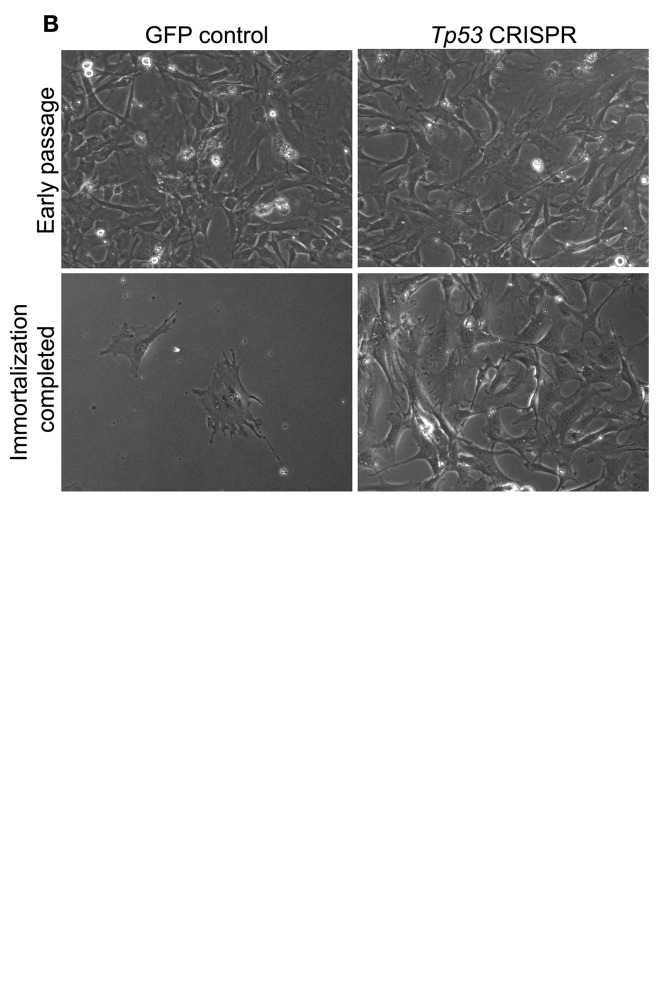
Representative images of mouse embryonic fibroblasts (MEFs) after electroporation and throughout the process of immortalization. A. GFP channel and phase contrast images of cells transfected with the GFP control or the *Tp53* CRISPR constructs 16 h after electroporation. B. Representative phase contrast images of cells transfected with the GFP control or *Tp53* CRISPR constructs. Top panels: cells imaged four days after electroporation, before differences in the cell proliferation rate are detectable between the two conditions. Bottom panels: Cells imaged 17 days after electroporation and after being split twice. Cells transfected with the GFP control construct underwent senescence (as indicated by the larger size) or died. In contrast, cells transfected with the *Tp53* CRISPR constructs proliferate robustly, indicating successful immortalization. Scale bar: 50 μm. Magnification: 10×.

20. Evaluate the electroporation efficiency by observing the expression of GFP in the GFP control group under an inverted fluorescence microscope 16–24 h after electroporation. Some cell death (20%–60%, depending on the electroporation program used) is expected after electroporation. >30% of the healthy cells should show GFP expression (Figure 3A).

21. Sixteen to twenty-four hours after electroporation, change culture media to complete cell culture media (with penicillin-streptomycin).

22. Continue to culture and passage the cells when they reach confluency (Figure 3B, top panel) (we typically do a 1:15 split) until the cells in the GFP control group have died off (usually within 2–3 weeks). Cells from the *Tp53* CRISPR group should continue to proliferate, which indicates successful immortalization (Figure 3B, bottom panel).

23. Freeze down the established iMEFs and/or culture for further experiments.

*Note: All waste from tissue culture procedures should be disposed of through approved biohazard protocols.*

## Validation of protocol

We have successfully used this protocol to generate more than 15 independent iMEF lines, including those described in Griffiths et al. (Figures 3–5 and associated supplementary data figures) [16].

## General notes and troubleshooting

**Troubleshooting**

**Table:** 

Problem	Possible cause	Solution
Low cell viability after establishing MEFs	The trypsin digestion is too long.	Adjust the trypsinization time and neutralize the trypsin as soon as the incubation is over.
	The dissected embryos are over-homogenized.	Reduce the number of times that the dissected embryos are pipetted up and down. The homogenization step should be gentle.
	The MEF isolation procedure takes too long.	Get familiar with the procedure and have all solutions and supplies ready before starting.
Low cell viability after thawing MEFs	Cells are not healthy or have already undergone senescence.	If the freshly isolated MEFs from each embryo do not reach confluency within 3 days, or if excessive death is observed on the day after isolation, the culture should be discarded, and the MEF isolation should be repeated from new embryos.
Low cell viability after electroporation	Poor cell health before electroporation.	The cells must be healthy and proliferating robustly before electroporation.
	DNA concentration is not optimal for the cells being electroporated.	The concentration of DNA used for the electroporation should be optimized. Try reducing the DNA concentration.
	Microbubbles in the Neon pipette tip.	Ensure that the Neon tip is firmly fitted onto the Neon pipette. Press the push button down to the first stop fully before inserting the tip into the cell/DNA master mix to withdraw the sample. Avoid introducing bubbles while mixing the cell/DNA suspension.
	The electroporation program is too harsh for the cells.	Try different electroporation parameters.
	The electroporation buffer is not optimal for the cells	Other electroporation buffers, such as Buffer R (from the Neon kit) can be used, though we have found the TransIT-LT1 transfection reagent to perform better.
	Endotoxin contamination in the DNA preparation.	Use an endotoxin-free purification kit for DNA isolation.
Low electroporation efficiency	Electroporation parameters are too weak.	Optimize by increasing pulse voltage, width, and number (see page 34 of the Neon transfection user guide: https://assets.thermofisher.com/TFS-Assets/LSG/manuals/neon_device_man.pdf).
	Cells are not proliferating robustly prior to electroporation.	Ensure that the cells are within 0–3 passages and are proliferating robustly. Also, ensure that cells have not undergone senescence. (Cells in senescence usually appear larger.)
	DNA preparations have salt contamination or other impurities.	Use a high-quality purification kit for DNA isolation. Wash the DNA pellets with 70% ethanol during isolation to remove salt.
